# Measurement precision and biological variation of cranial arteries using automated analysis of 3 T magnetic resonance angiography

**DOI:** 10.1186/1129-2377-15-25

**Published:** 2014-05-07

**Authors:** Faisal Mohammad Amin, Elisabet Lundholm, Anders Hougaard, Nanna Arngrim, Linda Wiinberg, Patrick JH de Koning, Henrik BW Larsson, Messoud Ashina

**Affiliations:** 1 Danish Headache Center and Department of Neurology, Glostrup Hospital, Faculty of Health and Medical Sciences, University of Copenhagen, Nordre Ringvej 57, DK-2600 Glostrup, Denmark; 2 Division of Image Processing, Department of Radiology, Leiden University Medical Center, Leiden, Netherlands; 3 Functional Imaging Unit, Diagnostic Department, Glostrup Hospital, Faculty of Health and Medical Sciences, University of Copenhagen, Copenhagen, Denmark

**Keywords:** Magnetic resonance angiography, Middle meningeal artery, Middle cerebral artery, Migraine

## Abstract

**Background:**

Non-invasive magnetic resonance angiography (MRA) has facilitated repeated measurements of human cranial arteries in several headache and migraine studies. To ensure comparability across studies the same automated analysis software has been used, but the intra- and interobserver, day-to-day and side-to-side variations have not yet been published. We hypothesised that the observer related, side-to-side, and day-to-day variations would be less than 10%.

**Methods:**

Ten female participants were studied using high-resolution MRA on two study days separated by at least one week. Using the automated LKEB-MRA vessel wall analysis software arterial circumferences were measured by blinded observers. Each artery was analysed twice by each of the two different observers. The primary endpoints were to determine the intraclass correlation coefficient (ICC) and intra- an inter-observer, the day-to-day, and side-to-side variations of the circumference of the middle meningeal (MMA) and middle cerebral (MCA) arteries.

**Results:**

We found an excellent intra- and interobserver agreement for the MMA (ICC: 0.909-0.987) and for the MCA (ICC: 0.876-0.949). The coefficient of variance within observers was ≤1.8% for MMA and ≤3.1% for MCA; between observers ≤3.4% (MMA) and ≤4.1% (MCA); between days ≤6.0% (MMA) and ≤8.0% (MCA); between sides ≤9.4% (MMA) and ≤6.5% (MCA).

**Conclusion:**

The present study demonstrates a low (<5%) inter- and intraobserver variation using the automated LKEB-MRA vessel wall analysis software. Furthermore, the study also suggests that the day-to-day and side-to-side variations of the MMA and MCA circumferences are less than 10%.

## Background

The three-dimensional time-of-flight magnetic resonance angiography (3D-TOF-MRA) method has enabled *in vivo* investigation of the human cranial arteries with a relatively high spatial resolution. The 3D-TOF-MRA method is simple to use and requires no intravenous contrast to visualize the arteries [1]. Thus, it is possible to detect changes of the luminal size of larger and smaller arteries relatively precise. This method has been employed in several headache and migraine studies to measure arterial changes before and after infusion of different vasoactive drugs and during versus outside attacks of migraine headache [2-8]. Most recently, using this method we compared the arterial circumferences on the headache side with the non-headache side and a migraine attack day with a non-headache day in patients with migraine. Surprisingly, we found intracranial but *not* extracranial arterial dilatation on the headache-side relative to the non-headache-side [6]. In a previous MRA study of drug-induced migraine attacks the middle cerebral artery (intracranial) and middle meningeal artery (extracranial) were both dilated on the pain-side versus the non-pain side [4], while another MRA study of drug-induced migraine attacks reported no side-to-side changes at all [2]. Although, the differences were ascribed different drug effects, it raised the question about the biological variations in day-to-day and side-to-side arterial circumference in migraine patients. In addition, these variations may also be affected by the observer related variability. Automated analysis of the acquired MRA images may reduce observer related variability and may also ensure better repeatability across different studies. The LKEB-MRA vessel wall analysis software [9], which has been used in several headache and migraine studies [2-8], provides an automated method to detect the vessel lumen contour accurately. The required user interaction is limited to placing only a proximal and a distal point in the vessel of interest [9]. In the present study, we therefore initially investigated the intra- and inter-observer variations and then the day-to-day, and side-to-side variations of the MMA and MCA using LKEB-MRA vessel wall analysis software [9]. We hypothesized that the observer related variability would be less than 5% using automated analysis software and the biological variations, including observer variation, would be less than 10%.

## Methods

### Participants

This study included 10 female migraine patients without aura who were recruited between July 2011 and February 2012 via a Danish website for recruitment of participants for biomedical research projects ( http://www.forsoegsperson.dk). Exclusion criteria were: a history of neurological disorder (except migraine without aura or infrequent tension-type headache less than 5 days per month), a history of cardiovascular disease, any daily medication intake (except oral contraceptives), any other somatic or psychiatric disease, pregnant or breast feeding, and any contraindication for magnetic resonance imaging. The regional Ethical Committee of Copenhagen (Denmark) approved the study. All participants gave their written informed consent and the study was conducted in accordance with the Helsinki Declaration. This study was a part of a larger study investigating physiological effects of vasoactive drugs [8].

### Study design

Magnetic resonance angiography (MRA) was performed in all participants on two different study days with at least one week between the study days. None of the participants had a headache or an intake of any type of medication during or 48 h prior to the MRA scans. All participants were abstinent from tobacco, alcohol and caffeine-containing food or drinks for 8 h, and totally fasting in 4 h prior to the MRA scans. Moreover, heart rate, blood pressure, respiratory frequency, end-tidal pressure of carbon dioxide (CO_2_), haematocrit, and haemoglobin levels were measured on both days.

### MRA acquisition

We used a 3.0 Tesla Philips Achieva machine (Philips Medical Systems, Best, Netherlands) with an eight-element phased-array receiver head coil to acquire single-slab three-dimensional time-of-flight MRA of the middle cerebral artery (MCA [FOV, 200 × 200 × 74 mm^3^; matrix size, 800 × 406; acquired voxel resolution, 0.25 × 0.49 × 1.00 mm^3^; reconstructed resolution, 0.20 × 0.20 × 0.50 mm^3^; TR, 25 ms; TE, 3.5 ms; flip angle 20°; sense factor 2; four chunks; acquisition time, 9 min 3 seconds]) and the middle meningeal artery (MMA [FOV, 200 × 200 × 16 mm^3^; matrix size, 800 × 571; acquired voxel resolution, 0.25 × 0.35 × 0.70 mm^3^; reconstructed resolution, 0.20 × 0.20 × 0.35 mm^3^; TR, 25 ms; TE, 3.5 ms; flip angle 20°; sense factor 3; four chunks; acquisition time, 5 min29 seconds]). We used the MCA location and the branching point of the MMA as references to plan the MRA slabs on the same position on both days. It would have been most optimal to use the same scan for both arteries, but as the MMA is smaller than MCA, small circumference changes would not have been detected. We therefore used a smaller voxel size for the MMA. However, if the MMA scan was used to record both MMA and MCA it would have consumed much more time, increasing the risk of movement artefacts. Even though, we used a higher resolution for the MMA scan it was not enough to capture the intracranial part of the MMA, because the artery size decreases and vary much in location inside the cranial cavity.

### Data analysis

**Figure 1:**
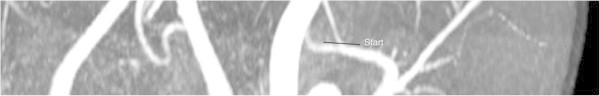
**The start of the middle meningeal artery segment was selected by marking the branch from the main trunk of the maxillary artery.** The black line indicates the start point.

**Figure 2:**
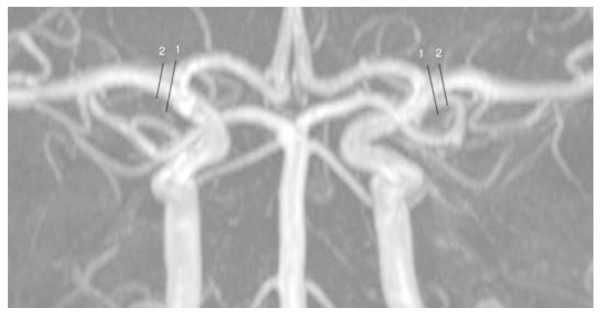
**The start point of the middle cerebral artery (MCA) segment was identified at the point where artery branches from the main trunk of the internal carotid artery.** It is difficult to choose where exactly the MCA starts. The black lines marked with 1 and 2 show two different start points. If it is chosen that MCA starts from line number 1, the MCA in every analysis in this patients should start from number 1.

The MRA data were transferred from the scanner computer to a remote workstation in DICOM format and then analysed by the LKEB-MRA vessel wall analysis software (version 6.2007). The MMA was identified by marking the branch from the main trunk of the maxillary artery (Figure 1). The MCA was identified by marking the branch from the main trunk of the internal carotid artery (Figure 2). The software calculated a path line and measured the diameter and circumference of the selected vessel segment every 0.2 mm perpendicular to the centre line, from which the average circumference of a 5 mm long vessel segment was finally obtained. To determine intra- and inter-observer variations, all images were analysed twice by two different investigators, who were blinded to the experimental day. Each observer analysed a total of 40 MMAs (EL and LW) and 40 MCAs twice (NA and LW) (10 right-sided and 10 left-sided arteries for day 1 and 2). The only manual input by the observer was selection of a start and an end point on each image (Figures 1 and 2). The results for the investigator who had the best intra-observer variation were used to analyse the biological variations.

### Statistics

All absolute values are presented as mean (SD). Age, height, weight, and days between the experiments are presented as mean (range).

The primary endpoints of the study were to determine the 1) intra-observer, 2) inter-observer, 3) day-to-day, and 4) side-to-side variations of the circumference of the MCA and the MMA.

We initially assessed the intra- and inter-observer measurement reliability by determining the Intraclass Correlation Coefficient (two-way mixed; absolute agreement). Coefficient of variance was then determined for all four endpoints using the Cfvar function in IBM SPSS Statistics software (version 20). We tested for differences in mean arterial circumferences within observers and between observers, days and sides using the paired samples *t* test. Differences in the physiological and biochemical data were also tested using the paired samples *t* test.

We used IBM SPSS Statistics (version 20) for all statistical analyses. No adjustment for multiple analyses was made. Thus, the level of significance at 0.05 was accepted for each comparison.

## Results

All ten participants (10 female [6 right-handed, 4 left-handed], mean age 24 [range 19–31], height 165 cm [range 157–174], weight 58 kg [range 52–68] were scanned on two different experimental days (mean time between the scans 17 days [range 7–29]). There was no difference in the heart rate, the respiratory frequency, end-tidal pressure of CO_2_, hematocrit, and hemoglobin levels between the two days. However, the blood pressure was slightly higher on the first day (Table 1).

**Table 1:** Mean values (±SD) of physiological variables between the two days

**Variable**	**Day 1**	**Day 2**	***P*-value**
Heart rate (beats/min)	63 (5)	64 (5)	0.250
Systolic blood pressures (mmHg)	121 (10)	116 (11)	0.028
Diastolic blood pressure (mmHg)	68 (8)	65 (8)	0.093
Mean arterial blood pressure (mmHg)	85 (7)	82 (8)	0.038
Respiratory frequency (breaths/min)	15 (3)	15 (3)	0.343
End-tidal pressure of CO2 (kPa)	4.8 (0.2)	4.7 (0.3)	0.348
Hematocrit	0.38 (0.03)	0.39 (0.03)	0.333
Hemoglobin (mmol/L)	8.1 (0.5)	8.2 (0.5)	0.264

*P*-value: The paired samples *t*-test.

### Intra- and inter-observer agreements and variations

The intraclass correlation coefficients for average measures were excellent for the MMA and the MCA measurements. MMA: EL 0.936 (95% CI 0.879 to 0.966) and LW 0.985 (95% CI 0.971 to 0.992). MCA: NA 0.949 (95% CI 0.904 to 0.973) and for LW 0.938 (95% CI 0.884 to 0.967).

**Figure 3:**
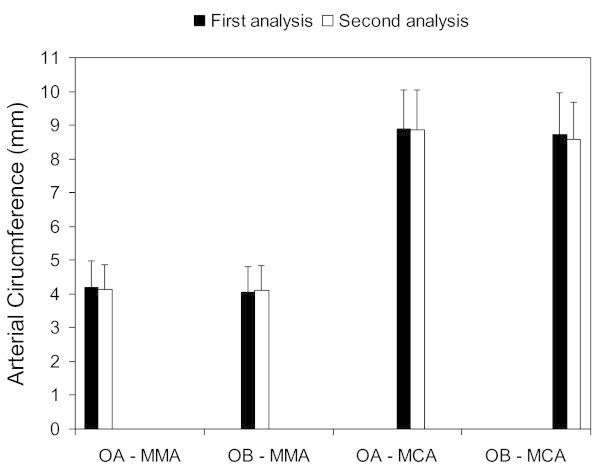
**Intra- and inter-observer variations in the middle meningeal (MMA) and middle cerebral (MCA) arteries.** Mean arterial circumferences (mm) are shown for the first (dark columns) and second (white columns) analyses done by observer A (OA) and observer B (OB). Bars represent the standard deviations.

The coefficient of variance was smaller in the MMA (observer EL, 1.8% and observer LW, 1.8%) than in the MCA (observer NA, 3.0% and observer LW, 3.1%) (Figure 3).

The agreement between observers was also very high for the MMA measurements (first 0.909 (95% CI 0.827 to 0.952) and second 0.987 (95% CI 0.976 to 0.993)). The MCA measurements also showed high interobserver agreement (first 0.876 (95% CI 0.768 to 0.934) and second 0.929 (95% CI 0.827 to 0.967). We found a smaller coefficient of variation when we compared an observer’s second analysis with the other observer’s second analysis (MMA, 1.7% and MCA, 3.3%) compared to the variance in their first analyses (MMA, 3.4% and MCA, 4.1%) (Figure 3).

### Day-to-day variations

The right MMA circumference varied 6.0% and left 5.9% between the two days. There was an 8.0% variation in the right MCA circumference and 7.1% in the left sided MCA circumference (Table 2).

**Table 2:** Day-to-day differences of the mean arterial circumference (±SD) and the coefficient of variance

**Artery**	**Side**	**Day 1**	**Day 2**	***P*-value**	**Variance**
MMA	Right	4.17 mm (0.77)	4.03 mm (1.00)	0.327	6.0%
	Left	4.18 mm (0.54)	4.15 mm (0.65)	0.810	5.9%
MCA	Right	9.35 mm (1.36)	8.68 mm (0.80)	0.106	8.0%
	Left	8.83 mm (1.44)	8.61 mm (0.94)	0.583	7.1%

MMA = middle meningeal artery; MCA = middle cerebral artery; *P*-value: The paired samples *t*-test.

### Side-to-side variations

We found almost the same variations between the right- and left-sided MMAs and MCAs on both experimental days. On day 1 MMA varied 9.3% and 9.4% on day 2. The coefficient of variance for the MCA was 6.4% on day 1 versus 6.5% on day 2 (Table 3).

**Table 3:** Side-to-side differences of the mean arterial circumference (±SD) values and the coefficient of variance in 10 subjects

**Artery**	**Day**	**Right**	**Left**	***P*-value**	**Variance**
MMA	1	4.17 mm (0.77)	4.18 mm (0.54)	0.958	9.3%
	2	4.03 mm (1.00)	4.15 mm (0.65)	0.613	9.4%
MCA	1	9.35 mm (1.36)	8.83 mm (1.44)	0.155	6.4%
	2	8.68 mm (0.80)	8.61 mm (0.94)	0.076	6.5%

MMA = middle meningeal artery; MCA = middle cerebral artery; *P*-value: The paired samples *t*-test.

## Discussion

This is the first MRA based study of experimental and biological variations in the circumference of human middle cerebral and meningeal arteries. Using the automated LKEB-MRA vessel wall software [9], we found an excellent intra- and interobserver agreements and relatively small variations within (<3.1%) and between (<4.1%) observers, as well as between the days (<8.0%) and sides (<9.4%).

### Observer variations

The variation in the MCA was higher than in the MMA for both observers. Identification of the start points are complicated by the anatomy of the vessel. An identical start point of the MMA is more plausible to determine consistently as there is a marked decrease in vessel size where the MMA branches out from the maxillary artery (Figure 1). However, in some cases the MMA branches directly from the external carotid artery, where a starting point is more difficult to select. On the contrary, the size difference between the distal part of the internal carotid artery and the MCA very small, which makes it challenging to identify the exact same starting point within and between observers (Figure 2). Unlike the main trunk of the extracranial MMA, the MCA circumference decreases along its main trunk, making it more susceptible to differences in the starting point. For instance, if the start is selected 2–3 mm more distally on one image, it results in a smaller circumference of that MCA.

The variation of the second analyses was almost the same between the observers as it was within the observers. In contrast, comparison of the first analyses showed varied slightly more, indicating that some exercise is necessary to analyse the arteries accurately. Observers in this study (EL, LW, NA) had no previous experience with the software or analysis of MRA images. They received the same 15 min instruction by an experienced observer (FMA) before they started the measurements. The gold standard to assess MCA diameter changes before the advent of MRI was the contrast agent based arteriography. Intravenous contrast based methods are not optimal for repeated measurements. Therefore, many studies of vascular mechanisms of drug-induced and spontaneous headache and migraine used the transcranial Doppler (TCD) method. The TCD methods is based on recordings of the velocity changes of the MCA, which can be calculated to diameter changes given that the cerebral blood flow is constant [10]. However, the variations of the TCD methods are expected to be large due to several factors, such as different angles of insonation, heart rate variability, and those caused by different observers (13%) [11]. To the best of our knowledge, no such study of the MMA exits.

### Biological variations of the cranial arteries

**Figure 4:**
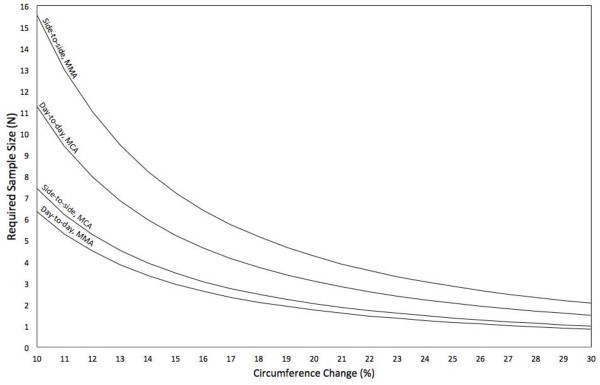
**Sample size determination using the coefficient of variance for unpaired data in 3D-TOF-MRA studies where day-to-day and side-to-side differences of the middle meningeal (MMA) and middle cerebral (MCA) arterial calibre are desired (5% significance, 90% power).** For paired data, the sample size is half of the required sample size should be used: N = 21*(coefficient of variance)^2^ / (lnμ_0_ – lnμ_1_)^2^.

The day-to-day and side-to-side variations in the MCA circumference found in the present study were smaller than the previously reported variation in the blood velocity (BV) of MCA using TCD [11]. An increase in the BV indicates vasoconstriction, whereas decreased velocity represents increased vessel diameter, provided that the cerebral blood flow is constant. Different factors have been demonstrated to influence the BV and thereby the day-to-day variation in the vessel size. These factors include age [12-16], haematocrit [17], and the end-tidal pressure of CO_2_[18]. In addition, intake of alcohol or caffeine-containing food or drinks, use of medication, and headache [6,19] may also play a role in the day-to-day variation. The age is unlikely to play any role in the present study, as all participants rescanned within 30 days (mean 17 days). The haematocrit and end-tidal pressure of CO_2_ were determined on both days and showed no statistical differences between the days in our study, suggesting a minimal input to the day-to-day differences seen in the present study. The participants were abstinent from tobacco, alcohol, and caffeine for at least 8 hours, and they had no medication use or headache for 48 h prior to the experiment starts. While there was a small difference in the day-to-day variation, the side-to-side differences were almost same on both days. The side-to-side differences may be explained by anatomical differences of the arteries. These data provide a possibility to calculate sufficient sample size in future studies (Figure 4). However, these data also suggest that only circumference changes higher than 10% in headache and migraine studies can be considered clinical relevant using this method.

### Methodological considerations

Although, the present day-to-day variations are relatively small, there are some methodological considerations, which have to be mentioned. First, we are not able to exactly sort out how much of the found biological variation is real and how much is influenced by the observer, but it is certainly less than 10%. However, the 3D-TOF-MRA method itself can affect the measurements (i.e. two MRA's obtained in the same scan session, may also play a role). Even small body movement during the scan can result in blurred MRA images. Blurred images makes the arteries appear larger, but at the moment, there is no way to detect the image quality for motion of the 3D-TOF-MRA other than visual assessment by the observer or calculation of signal-to-noise ratios, when comparing two different MRA images. Another important consideration when comparing different segments with each other is that signal obtained from arteries located in-plane decrease the more distally the selected segments are. It is therefore crucial that exact the same segment is selected across different images, when comparison between two days or conditions is desired. A possible difference between the MCA and MMA variations could also be related to the fact that the MMA is running along the plane of acquisition with possible increased risk of flow artefacts.

## Conclusion

The present study demonstrates a low (<5%) inter- and intraobserver variation using the automated LKEB-MRA vessel wall analysis software. Furthermore, the study also suggests that the day-to-day and side-to-side variations of the MMA and MCA circumferences are less than 10%.

## Abbreviations

3D-TOF: Three-dimensional time-of-flight; BV: Blood velocity; CO_2_: Carbon monoxide; DICOM: Digital Imaging and Communcations in Medicine; FOV: Field-of-view; MCA: Middle cerebral artery; MMA: Middle meningeal artery; MRA: Magnetic resonance angiography; SD: Standard deviation; TCD: Transcranial Doppler; TE: Echo time; TR: Repetition time.

## Competing interests

The authors declare that they have no competing interests in relation to this study.

## Authors’ contributions

FMA and AH carried out the scans, designed the study, performed statistical tests, and drafted the manuscript. EL, NA, and LW analysed the images, were involved in study design, and participated in manuscript drafting. PJHdK and HBWL were involved in interpretation of data and revising the manuscript critically for important intellectual content. MA designed the study, was involved in interpretation and drafting, and revised the manuscript critically. All authors have given final approval of the version to be published.
